# Degradation of Reactive Blue 19 (RB19) by a Green Process Based on Peroxymonocarbonate Oxidation System

**DOI:** 10.1155/2021/6696600

**Published:** 2021-03-03

**Authors:** Thi Bich Viet Nguyen, Ngan Nguyen-Bich, Ngoc Duy Vu, Hien Ho Phuong, Hanh Nguyen Thi

**Affiliations:** ^1^Faculty of Chemistry, Hanoi National University of Education, 136 Xuan Thuy, Cau Giay, Hanoi 10000, Vietnam; ^2^Faculty of Chemistry, VNU University of Science, Vietnam National University, 19 Le Thanh Tong, Hoan Kiem, Hanoi 10000, Vietnam; ^3^Research Center for Environmental Technology and Sustainable Development, VNU University of Science, Vietnam National University, 334 Nguyen Trai, Thanh Xuan, Hanoi 10000, Vietnam; ^4^Department of Chemistry, Hanoi Pedagogical University 2, 32 Nguyen Van Linh, Phuc Yen, Vinh Phuc 15000, Vietnam

## Abstract

The effectiveness of peroxymonocarbonate (HCO_4_^−^) on the degradation of Reactive Blue 19 (RB19) textile dye was investigated in this study. The formation kinetics of HCO_4_^−^ produced *in situ* in a H_2_O_2_ − HCO_3_^−^ system was studied to control the experimental conditions for the investigation of RB19 degradation at mild conditions. The effects of metallic ion catalysts, the pH, the input HCO_3_^−^ and Co^2+^ concentrations, and UV irradiation were studied. The obtained result showed that Co^2+^ ion gave the highest efficiency on accelerating the rate of RB19 degradation by the H_2_O_2_–HCO_3_^−^ system. In the pH range of 7–10, the higher pH values resulted in faster dye degradation. The reaction orders of the RB19 degradation with respect to Co^2+^ and HCO_3_^–^ were determined to be 1.2 and 1.7, respectively. The UV irradiation remarkably enhanced the radical formation in the oxidation system, which led to high degradation efficiencies. The COD, TOC removal, and HPLC results clearly revealed complete mineralization of RB19 by the H_2_O_2_ − HCO_3_^−^−Co^2+^ system.

## 1. Introduction

Reactive dyes play an important role in textile industries. However, they may cause serious environmental problems as they are often nonbiodegradable and toxic to aquatic systems due to their complex aromatic molecular structures [1]. Once being discharged to the environment without destructive treatment, these water-soluble dyes can remain for a long time and alter the quality of water bodies by preventing light penetration and hindering photosynthesis, thereby affecting the ecosystems [2]. Among reactive dyes, anthraquinone dyes contain reactive groups with reinforced structures, which makes them difficult to degrade naturally and can be bioaccumulative in animals [3]. For example, an estimated half-life of C.I. Reactive Blue 19 at pH 7.0 and 25°C based on kinetic studies is *ca*. 46 years [4]. The concern over the ecotoxicity of these reactive dyes has led to the need to develop more efficient methods for their remediation from industrial effluents before discharging to water bodies. Textile wastewater treatment is not only decolorizing but also degrading and mineralizing dye molecules.

Among technologies for dye treatment, advanced oxidation processes (AOPs) have attracted increasing attention due to their efficiency and ability to thoroughly remove pollutants from wastewater effluents. These AOPs base on the strong activity of *in situ* formed free radicals, among which the most common radical is ^•^OH with a redox potential of +2.8 V. Thanks to their strong oxidizing capacity, organic substances can be completely mineralized into CO_2_ and H_2_O. Some typical processes for active radical generation include Fenton, peroxone, H_2_O_2_/UV, O_3_/UV, and TiO_2_/UV.

A photochemical-based AOP of textile wastewater containing RB19 dye has been studied using the UV/K_2_S_2_O_8_ system [5]. The results showed that only 50% of RB19 dye was removed after 5 hours in the dark system, while the UV-irradiated one gave a complete color removal in less than 30 min and 78.5% COD has been removed after 3 hours of irradiation time. The decolorization rate was fitted to the *pseudo*-first-order kinetic model regarding dye concentration. Another study on UV irradiation effect on RB19 decolorization conducted by Tehrani et al. who compared the RB19 decolorization efficiency by ozonation and UV-enhanced ozonation revealed that a UV irradiation by mercury lamp only increased COD removal efficiency, not the decolorization efficiency [6].

In advanced oxidation processes, besides ^•^OH, ^•^O_2_^−^, and ^1^O_2_ radicals, other oxidizing agents such as HCO_4_^−^, ^•^CO_3_^−^, and HCO_3_^•^ also play a crucial role in oxidation performance [7]. Peroxymonocarbonate (HCO_4_^–^) ion is known to be an active oxidant in the oxidation of organic compounds [8, 9]. This oxidizing agent was proved to have 100–500 times stronger reactivity in sulfide organic oxidation compared to hydrogen peroxide [10]. Although ^•^CO_3_^−^ and HCO_3_^•^ radicals are reported to be less reactive than ^•^OH, their occurrence at higher concentration and longer lifetime may provide sufficient oxidation potential. Another advantage of ^•^CO_3_^−^ and HCO_3_^•^ radicals is a simple production technique.

One of the major benefits of the oxidation process based on the peroxymonocarbonate system is its greenness toward the environment. As it can be prepared by mixing H_2_O_2_ with HCO_3_^−^ or CO_3_^2−^, the oxidation process does not require neither expensive nor toxic chemicals nor energy demand. Furthermore, it does not generate secondary sludge waste or require chemicals to adjust pH as compared to the Fenton process.

The aim of this work was two‐fold: (i) to evaluate the effectiveness of *in situ* generated HCO_4_^−^ oxidizing agent in the HCO_3_^−^−H_2_O_2_ system in degrading the RB 19 textile dye under different experimental conditions and (ii) to verify the products of RB19 degradation by oxidation system based on HCO_4_^−^.

## 2. Materials and Experimental Procedure

### 2.1. Chemicals and Apparatus

Reactive Blue 19 dye was purchased from Sigma-Aldrich. Other chemicals and reagents used in this study were of analytical grade and used as received without any further purification.

The RB19 degradations were monitored by measuring RB19 absorbance at 588 nm using a Biochrom Libra S60 UV-Vis Spectrophotometer. The degradation efficiency was calculated using the following equation: RB19 degradation (%) = (*C*_o_ − *C*_t_)/*C*_o_ × 100%, where *C*_o_ and *C*_t_ are the initial and remaining RB19 concentrations (ppm) at time *t* (min), respectively. These concentrations were determined using the standard curve **Abs** = (8.4 ± 0.1).10^3^**C**_**RB19**_ (mg/L) (*R*^2^ = 0.999) with the LOD and LOQ of 0.7 and 2.3 mg/L, respectively. pH values were measured by a Lab850 pH meter (with BlueLine 14 pH electrode). COD values were determined by the oxidation standard method [11]. Total carbon and total inorganic carbon were measured on a multi-N/C 2100 TOC analyser (Analytik Jena AG). A Shimadzu RP-HPLC equipped with a PDA-M20A detector was used to investigate the products of the degradation. A mixture of acetonitrile and phosphate buffer at pH of 4.7 (50/50, v/v) was used as a mobile phase with a flow rate of 1 mL/min, an injection volume of 20 *μ*L, and an oven temperature of 40°C.

### 2.2. Experimental Procedure

#### 2.2.1. *In Situ* Peroxymonocarbonate Preparation

Peroxymonocarbonate (HCO_4_^−^) solutions were prepared *in situ* by mixing sodium bicarbonate with hydrogen peroxide at a molar ratio of 1 : 2 at ambient temperature. Kinetics of HCO_4_^−^ formation was evaluated by monitoring HCO_4_^−^ concentration at different times in three hours. In this experiment, HCO_4_^−^ was analyzed by a modified iodometric titration method [12]. In which, HCO_4_^−^ was first allowed to react with iodide at low temperature (below −10°C) to prevent the effect of H_2_O_2_. The formed I_2_ was then titrated with thiosulfate using a starch indicator.

#### 2.2.2. Degradation of RB19 by *In Situ* Generated HCO_4_^−^ in the H_2_O_2_ − HCO_3_^−^ System

**Figure 1:**
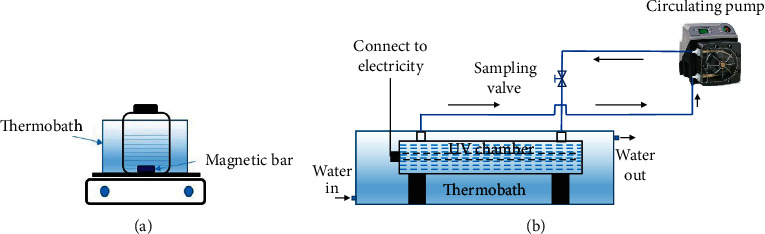
The reaction system (a) and the continuous UV-irradiated system (b) for the RB19 degradation.

The effects of different parameters on the RB19 degradation were investigated as described in Table 1 including the metallic ion catalyst (trials 1–9), the pH (trials 10–13), the HCO_3_^−^ concentration (trials 14–18), the Co^2+^ catalyst concentration (trials 19–22), and UV irradiation (trials 24–28). The RB19 concentration was 100.0 mg/L in all trials. Each trial was replicated three times. In trials 1–5, 8–22, and 26, the mixture of HCO_3_^−^ and H_2_O_2_ with a molar ratio of 1 : 2 was prepared in 50 minutes before adding to the RB19 solution to conduct the dye decolorization. For trials 1–23, the reactions were conducted in a 250 mL batch reactor controlled at 26 ± 1°C using a thermobath and thoroughly mixed by a magnetic stirrer as depicted in Figure 1(a). For trials 24–28, the reaction solutions were irradiated with the UVC light (254 nm, 12 W) in the UV chamber regulated by a thermobath as described in Figure 1(b). The flow speed through the UV chamber was maintained at 100 mL/min using a circulating pump. Dye decolorization was monitored by sampling at certain intervals and measuring light absorbance at the wavelength of 592 nm.

## 3. Results and Discussion

### 3.1. Formation of Peroxymonocarbonate

The peroxymonocarbonate ion was produced *in situ* by the reaction of HCO_3_^−^ and H_2_O_2_ according to the following reversible reaction [13]:

(1) $$\begin{array}{c} \overset{-}{\underset{3}{\text{HCO}}}+{\text{H}}_{2}{\text{O}}_{2}⇌\overset{-}{\underset{4}{\text{HCO}}}+{\text{H}}_{2}\text{O} \end{array}$$

**Figure 2:**
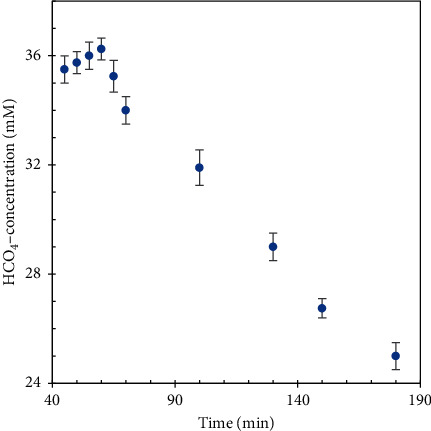
The variation of peroxymonocarbonate concentrations with time.

To determine the maximum formation of HCO_4_^−^ formed *in situ* in the system, the variation of HCO_4_^–^ concentrations with time at a HCO_3_^−^: H_2_O_2_ molar ratio of 1 : 2 ([HCO_3_^−^] = 312.5 mM) has been studied and the result is given in Figure 2. It is revealed that HCO_4_^−^ was formed at the beginning, reached a maximum concentration at around 50 minutes, and then slowly decreased. This phenomenon can be explained by the decomposition of HCO_4_^−^ after formation.

Therefore, the dye decolorization experiments by the HCO_4_^−^ agent in this study were carried out by mixing H_2_O_2_ with HCO_3_^−^ 50 minutes before adding to the dye solution.

### 3.2. RB19 Degradation by H_2_O_2_ − HCO_3_^−^ System

#### 3.2.1. Effect of Metallic Ion Catalysts

**Figure 3:**
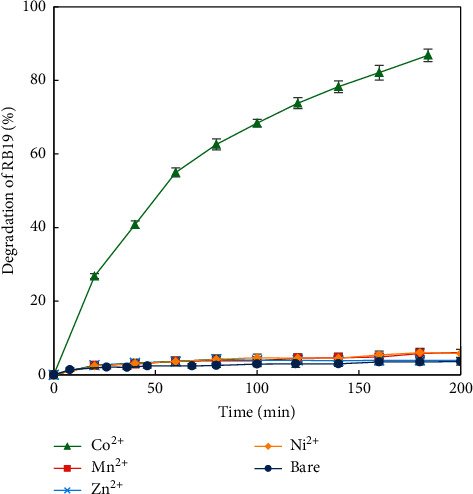
Degradation efficiency of RB19 *vs.* time without and with different metallic ion catalysts (100 mg/L RB19, 10 mM HCO_3_^−^, 20 mM H_2_O_2_, and 0.1 mg/L Ni^2+^/Mn^2+^/Zn^2+^/Co^2+^; pH 8).

The effect of metallic ion catalysts (*i.e.,* Ni^2+^, Mn^2+^, Zn^2+^, and Co^2+^) was investigated by performing trials 1–5 (Table 1). The result shown in Figure 3 reveals the most significant effect of Co^2+^ on the degradation of RB19 by the H_2_O_2_ − HCO_3_^−^ system. RB19 was degraded *ca.* 90% after 200 min in the presence of Co^2+^, while the similar systems with other metallic ions such as Mn^2+^, Zn^2+^, and Ni^2+^ and the bare system (without metallic ions) gave the degradation efficiency of less than 10%.

**Figure 4:**
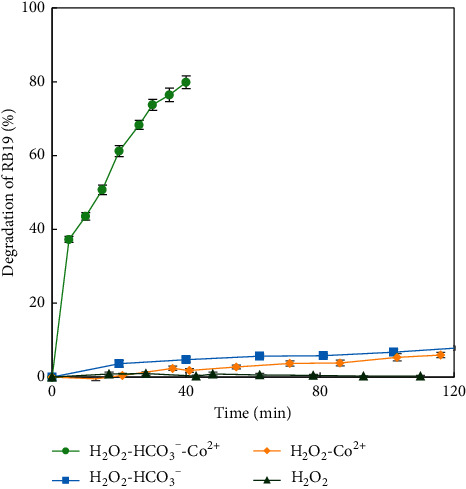
Comparison of the degradation of RB19 by H_2_O_2_, H_2_O_2_−Co^2+^, H_2_O_2_ − HCO_3_^−^, and H_2_O_2_ − HCO_3_^−^−Co^2+^ systems.

To clarify the role of Co^2+^ in the RB19 degradation by the HCO_4_^−^ agent, a comparison of RB19 degradation efficiencies by different systems (H_2_O_2_, H_2_O_2_−Co^2+^, H_2_O_2_ − HCO_3_^−^, and H_2_O_2_ − HCO_3_^−^−Co^2+^, trials 6–9) is presented in Figure 4. It clearly showed the remarkable effectiveness of Co^2+^ on the RB19 degradation of the H_2_O_2_-HCO_3_^–^ system. It can be seen from Figure 4 that the RB19 degradation efficiency is *ca.* 80% at a reaction time of 40 min with the H_2_O_2_ − HCO_3_^−^−Co^2+^ system, while three others (H_2_O_2_, H_2_O_2_−Co^2+^, and H_2_O_2_ − HCO_3_^−^) gave the degradation efficiencies of less than 10%. This phenomenon can be explained by the great capability of Co^2+^ in catalyzing oxidation reactions. In peroxyacid solutions, Co^2+^ enhances the decomposition of peroxy compounds to generate radicals [14, 15]. Applying the same mechanism, radicals are proposed to be formed by the following reactions:

(2) $$\begin{array}{c} \overset{-}{\underset{4}{\text{HCO}}}+{\text{CO}}^{2+}⟶\overset{2-}{\underset{3}{\text{CO}}}+{\text{Co}}^{3+}+{\text{OH}}^{*} \end{array}$$

(3) $$\begin{array}{c} \overset{-}{\underset{4}{\text{HCO}}}+{\text{Co}}^{2+}⟶\overset{•-}{\underset{3}{\text{CO}}}+{\text{Co}}^{3+}+{\text{OH}}^{-} \end{array}$$

Co^3+^ is then reduced to regenerate Co^2+^:

(4) $$\begin{array}{c} \overset{-}{\underset{4}{\text{HCO}}}+{\text{Co}}^{3+}⟶\overset{•}{\underset{4}{\text{CO}}}+{\text{Co}}^{2+}+{\text{H}}^{+} \end{array}$$

These radicals then react with the organic compounds through many steps and eventually form CO_2_ and H_2_O. The oxidation process of organic compounds occurs continuously with a crucial role of the Co^2+^/Co^3+^ redox couple.

#### 3.2.2. Effect of pH

**Figure 5:**
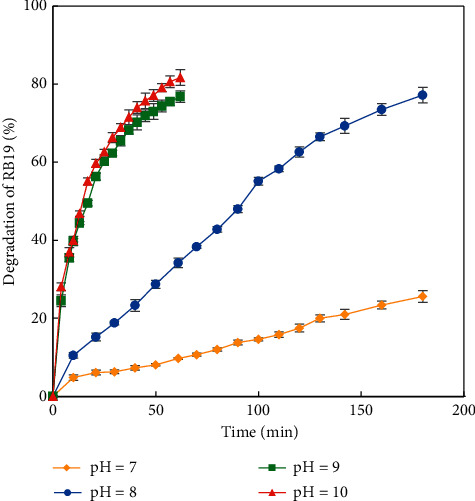
Degradation efficiency of RB19 *vs.* time at different pH values (100 mg/L RB19, 10 mM HCO_3_^−^, 20 mM H_2_O_2_, and 0.1 mg/L Co^2+^; pH 7, 8, 9, and 10).

The effect of pH was investigated by varying pH values from 7 to 10 and keeping constant other parameters (trials 10–13). The result shown in Figure 5 revealed that the increase in pH value causes faster dye degradation. However, the H_2_O_2_ − HCO_3_^−^ system acts as a buffer solution of pH *ca.* 8. Therefore, the pH value used during this study was adjusted to 8 in order to be the closest to the intrinsic pH of the solution.

#### 3.2.3. Effect of HCO_3_^−^ and Catalyst Concentrations: Kinetic Study of the RB19 Degradation Catalyzed by Co^2+^

To study the effect of HCO_3_^−^ and Co^2+^ catalyst concentrations on the RB19 degradation, as well as to study reaction kinetics, the RB19 degradation was carried out either at different HCO_3_^–^ concentrations (trials 14–18) and at different Co^2+^ concentrations (trials 19–22). The RB19 concentration decreased as a function of time.

**Figure 6:**
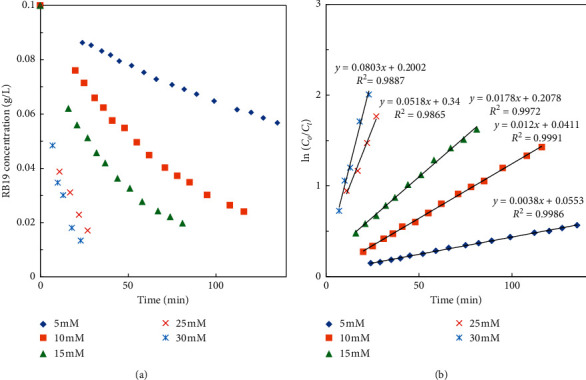
Dependence of (a) RB19 concentration and (b)ln(*Co*/*C*_*t*_)*vs*. time at different HCO_3_^−^ concentrations and 40 mM H_2_O_2_, 0.1 mg/L Co^2+^, and pH 8.

It can be seen from Figure 6(a) that RB19 degradation efficiency and rate remarkably increase with an increase in HCO_3_^−^ concentration from 5 mM to 30 mM while keeping constant other parameters (H_2_O_2_ and Co^2+^ concentrations, pH). It is because the increase in HCO_3_^−^ concentration leads to the increase in HCO_4_^−^ concentration according to the equilibrium reaction:

(5) $$\begin{array}{c} \overset{-}{\underset{3}{\text{HCO}}}+{\text{H}}_{2}{\text{O}}_{2}⇌\overset{-}{\underset{4}{\text{HCO}}}+{\text{H}}_{2}\text{O} \end{array}$$

**Figure 7:**
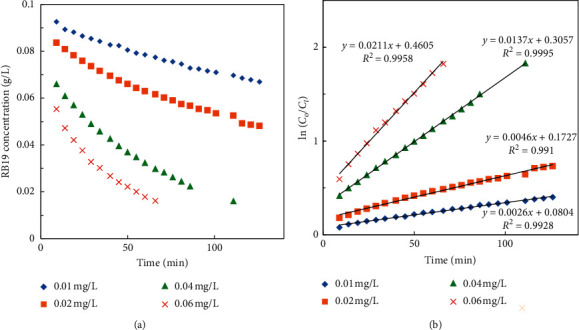
Dependence of (a) RB19 concentration and (b)ln(*Co*/*C*_*t*_)*vs.* time at different Co^2+^ concentrations and 10 mM HCO_3_^−^, 20 mM H_2_O_2_, and pH 8.

Similarly, Figure 7(a) shows a proportional relationship between the Co^2+^ concentration and the RB19 degradation efficiency and reaction rate. The reason for this may be the increase in Co^2+^ complexes as mentioned above.

The plots of ln(*Co*/*C*_*t*_)*vs.* time shown in Figures 6(b) and 7(b) reveal that the kinetic degradation of RB19 is well fitted with the first-order kinetic model given by the following equation:

(6) $$\begin{array}{c} \ln\left(\frac{Co}{C_{t}}\right)=k_{1}t. \end{array}$$

The reaction rate of RB19 degradation was assumed to be the *pseudo*-first-order kinetics with respect to RB19 as the following expression:

(7) $$\begin{array}{c} v=-\frac{\text{d}C_{t}}{\text{d}t}=k_{0}\left[\text{RB}19\right]{\left[\overset{-}{\underset{3}{\text{HCO}}}\right]}^{n1}{\left[{\text{H}}_{2}{\text{O}}_{2}\right]}^{n2}{\left[{\text{Co}}^{2+}\right]}^{n3}{\left[{\text{H}}^{+}\right]}^{n4}=k_{1}\left[\text{RB}19\right], \end{array}$$

where

(8) $$\begin{array}{c} k_{1}=k_{0}{\left[\overset{-}{\underset{3}{\text{HCO}}}\right]}^{n1}{\left[{\text{H}}_{2}{\text{O}}_{2}\right]}^{n2}{\left[{\text{Co}}^{2+}\right]}^{n3}{\left[{\text{H}}^{+}\right]}^{n4} \end{array}$$

is constant.

Integrating (7) gives (6): ln(*Co*/*C*_*t*_)=*k*_1_*t*.

The *pseudo*-first-order rate constants, *k*_1_ (min^−1^), were calculated from the slope of the plots of ln (*C*_o_/*C*_t_) *vs*. time *t* and subsequently used to calculate the experimental order of HCO_3_^−^ and Co^2+^.

*(1) Order of*HCO_3_^−^. To determine the order of HCO_3_^−^, experiments were conducted at different HCO_3_^−^ concentrations when [H_2_O_2_], [Co^2+^], and [H^+^] were kept constant.

From equation (8),

(9) $$\begin{array}{c} k_{1}=k_{2}{\left[\overset{-}{\underset{3}{\text{HCO}}}\right]}^{n1}, \end{array}$$

where *k*_2_=*k*_0_[H_2_O_2_]^*n*2^[Co^2+^]^*n*3^[H^+^]^*n*4^.

Taking the logarithm of both sides of (9) gives

(10) $$\begin{array}{c} \ln\;k_{1}=n_{1}\ln\left[\overset{-}{\underset{3}{HCO}}\right]+\ln\;k_{2}. \end{array}$$

*(2) Order of Co* ^*2+*^. Similarly, the order of Co^2+^ was determined when changing [Co^2+^] and keeping concentrations of other species constant.

(11) $$\begin{array}{c} \text{when}\left[{\text{H}}_{2}{\text{O}}_{2}\right],\left[\overset{-}{\underset{3}{\text{HCO}}}\right],\text{and}\left[{\text{H}}^{+}\right]\text{are constant,}\\ k_{1}=k_{3}{\left[{\text{Co}}^{2+}\right]}^{n3}, \end{array}$$

where *k*_3_=*k*_0_[HCO_3_^−^]^*n*1^[H_2_O_2_]^*n*2^[H^+^]^*n*4^.

Taking the logarithm of both sides of (11) gives

(12) $$\begin{array}{c} \ln\;k_{1}=n_{3}\ln\left[{\text{Co}}^{2+}\right]+\ln\;k_{3}. \end{array}$$

The experimental orders of HCO_3_^−^, *n*_1_, and Co^2+^, *n*_3_, were determined from the slope of the plot of ln(*k*_1_) *vs*. ln[HCO_3_^−^] and ln(*k*_1_) *vs*. ln[Co^2+^], respectively. The calculated data are shown in Table 2, and the result gives the experimental orders of HCO_3_^−^ and Co^2+^ of 1.7 and 1.2.

#### 3.2.4. Effect of UV Irradiation on the RB19 Degradation

The degradation of RB19 with the selected oxidation system (H_2_O_2_ − HCO_3_^−^ − Co^2+^) was carried out without and with UV irradiation (trials 5 and 28) compared to other systems (trials 6–8 and 23–27). The results are shown in Table 3. With UV irradiation, the RB19 degradation efficiency significantly increased (*ca*. 90%) in all other systems compared to the ones without UV irradiation (less than 5%). This is due to the fact that UV radiation plays a crucial role in creating the radical ^•^OH from H_2_O_2_ for the oxidation systems according to the photocatalytic mechanism as proposed in the literature [16]. Meanwhile, the highest RB19 degradation efficiency (97.6%) was obtained with the H_2_O_2_ − HCO_3_^−^−Co^2+^ system, slightly increased compared to the one without UV irradiation (79.9%). This strongly confirmed the degradation efficiency of the H_2_O_2_ − HCO_3_^−^−Co^2+^ system even without UV light.

### 3.3. Products of the RB19 Degradation

The COD values of the initial and final reaction solutions in which RB19 (0.1 g/L) was decomposed by the H_2_O_2_ − HCO_3_^−^−Co^2+^ system (after a reaction time of 60 min) were determined as 315 and 12.5 mg O_2_/L, respectively. The TOC (total organic carbon) value was obtained from the subtraction of TC (total carbon) and TIC (total inorganic carbon) values measured for the final solution, giving the result of 15.3 mg/L which is in accordance with the COD value (Table 4).

These results suggest a good mineralization of RB19 by the H_2_O_2_ − HCO_3_^−^−Co^2+^ system with a COD removal efficiency of 96%.

**Figure 8:**
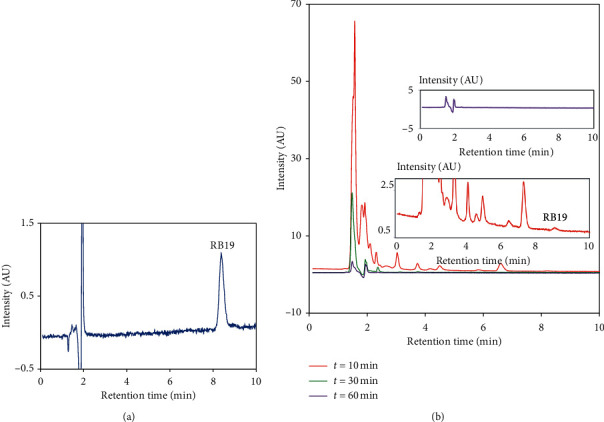
(a) The chromatogram of pure RB19 solution (1 mg/L); (b) the chromatograms of the RB19 degradation solution by the H_2_O_2_ − HCO_3_^−^−Co^2+^ system at different reaction times (*t* = 10, 30, and 60 min); the upper inset: the chromatogram recorded at a reaction time of 60 min; the lower inset: zoom-in view of the chromatogram recorded at a reaction time of 10 min.

Moreover, the RP-HPLC chromatograms of the RB19 degradation solutions using H_2_O_2_ − HCO_3_^−^−Co^2+^ system at reaction times of 10, 30, and 60 min were recorded at 280 nm (Figure 8(b)). It can be seen clearly that the intermediate products of RB19 degradation at a reaction time of 10 min and 30 min are less polar and eluted before the remained RB19 (zoom in the lower inset in Figure 8(b) compared with the chromatogram of pure 1 mg/L RB19 solution shown in Figure 8(a) with a retention time of 8.2 minutes). The unretained components are eluted at the time corresponding to the travel time of the mobile phase of 1.5 min. Most of these products are not observed after the reaction time of 30 min, and the chromatogram obtained for the reaction solution at 60 min is flattened (the upper inset). This result strongly supports the effectiveness of the RB19 degradation to a complete mineralization by the H_2_O_2_ − HCO_3_^−^−Co^2+^ system.

## 4. Conclusions

In the present work, the degradation efficiency of RB19 reactive dye has been proved to be strongly affected by the HCO_3_^−^ concentration and the presence of metallic ion catalysts, especially Co^2+^. This improvement can be explained by an enhancement in radicals generated by the Co^2+^/Co^3+^ redox couple. The *in situ* formation of peroxymonocarbonate ion HCO_4_^−^ is responsible for the oxidation efficiency of the system. This HCO_4_^−^ ion was determined to reach a maximum concentration after a H_2_O_2_ − HCO_3_^−^ mixing time of 40 min and remain stable for *ca.* 10 min. This kind of information is essential for practical applications of the system. The degradation kinetics of RB19 was determined to be the first order in RB19 dye while reaction orders of HCO_3_^–^ and Co^2+^ catalyst were 1.7 and 1.2, respectively. The products of RB19 degradation were investigated by RP-HPLC analyses and COD and TOC measurements, which suggest a complete mineralization of RB19 by the H_2_O_2_ − HCO_3_^−^−Co^2+^ system.

## Figures and Tables

**Table 1:** Conditions for RB19 decolorization by peroxymonocarbonate.

Trial	pH	[HCO_3_^−^] (mM)	[H_2_O_2_] (mM)	[M^2+^] catalyst (mg/L)	UV irradiation
1	8	10	20	—	No
2–5	8	10	20	0.1 (Ni^2+^, Mn^2+^, Zn^2+^, Co^2+^)	No
6	8	—	200	—	No
7	8	—	200	0.1 (Co^2+^)	No
8	8	100	200	—	No
9	8	100	200	0.1 (Co^2+^)	No
10–13	7, 8, 9, 10	10	20	0.1 (Co^2+^)	No
14–18	8	5, 10, 15, 25, 30	40	0.1 (Co^2+^)	No
19–22	8	20	40	0.01, 0.02, 0.04, 0.06 (Co^2+^)	No
23	8	—	—	—	No
24	8	—	—	—	Yes
25	8	—	20	—	Yes
26	8	—	20	0.1 (Co^2+^)	Yes
27	8	10	20	—	Yes
28	8	10	20	0.1 (Co^2+^)	Yes

**Table 2:** Experimental data for determining the orders of HCO_3_^–^ and Co^2+^ in RB19 degradation.

[HCO_3_^−^]	ln[HCO_3_^−^]	*k*_1_	ln*k*_1_	[Co^2+^]	ln[Co^2+^]	*k*_1_	ln*k*_1_
5	0.699	0.004	−2.420	0.01	−4.605	0.0026	−5.952
10	1.000	0.012	−1.921	0.02	−3.912	0.0046	−5.382
15	1.176	0.018	−1.750	0.04	−3.219	0.0137	−4.290
25	1.398	0.052	−1.286	0.06	−2.813	0.0211	−3.858
30	1.477	0.080	−1.095				
ln*k*_1_ = 1.7 ln[HCO_3_^–^]−3.6				ln*k*_1_ = 1.2 ln[Co^2+^]−0.5			

**Table 3:** The effect of UV irradiation on the RB19 degradation.

Oxidation system	RB19 degradation efficiency at 30 min reaction time (%)	
	Non-UV irradiation	UV irradiation
RB19	0.0	3.7 ± 0.3
RB19-H_2_O_2_	0.0	83.6 ± 3.6
RB19-H_2_O_2_-Co^2+^	1.8 ± 0.2	91.1 ± 2.9
RB19-H_2_O_2_-HCO_3_^–^	4.7 ± 2.7	96.7 ± 2.3
RB19-H_2_O_2_-HCO_3_^–^-Co^2+^	79.9 ± 3.5	97.6 ± 3.1

**Table 4:** Determination of COD and TOC of RB19 degradation.

	COD (mg O_2_/L)	TC (mg/L)	TIC (mg/L)	TOC (mg/L)
RB19 − H_2_O_2_ − HCO_3_^−^−Co^2+^ initial	315 ± 0.5	—	—	—
RB19 − H_2_O_2_ − HCO_3_^−^−Co^2+^ final	12.5 ± 0.3	141.5 ± 0.9	126.2 ± 0.9	15.3 ± 0.2

## Data Availability

The data used to support the findings of this study are available from the corresponding author upon request.
